# Visumax femtolasik versus Moria M2 microkeratome in mild to moderate myopia: efficacy, safety, predictability, aberrometric changes and flap thickness predictability

**DOI:** 10.1186/s12886-017-0520-5

**Published:** 2017-07-17

**Authors:** Magda A Torky, Yousif A Al Zafiri, Abeer M Khattab, Rania K Farag, Eman A Awad

**Affiliations:** 1 Department of ophthalmology, Faculty of medicine, Mansoura university, 24 Al-Gomhoria street, Mansoura, Egypt; 2 Department of ophthalmology, Dar AlShifa hospital, Kuwait city, Kuwait

**Keywords:** Visumax, Moria 2, myopia

## Abstract

**Introduction:**

This is an interventional prospective clinical study which was conducted to evaluate the efficacy, safety, predictability, ocular aberrations, and flap thickness predictability of Visumax femtosecond laser (FSL) compared to Moria M2 microkeratome (MK) in mild to moderate myopia.

**Methods:**

This study included 60 eyes who were divided into two groups. Thirty eyes in group (I) in which the flap was created with Visumax FSL, while in group II (30 eyes) the Moria M2 MK was used. Keratometric, refractive, and aberrometric measurements were compared preoperatively and 3 months postoperatively. The intraoperative subtraction pachymetry (the SP 100 Handy pachymeter (Tomey, Nagoya, Japan) was used for preoperative pachymetry and flap thickness measurement.

**Results:**

No significant difference was found between the two groups in regards to postoperative manifest sphere, spherical equivalent, astigmatism, safety indices nor ocular aberrations. Twenty six eyes (86.6%) in group I and 23 eyes in group II (76.6%) were within ±0.5D of the intended correction and 23 eyes (76.6%) in group I and 15 eyes in group II (50%) were within ±0.25D of the intended correction. In group I, the mean postoperative actual flap thickness was 100.12 ± 16.1 μm (81 to 122 μm), while in group II, it was 104.6 ± 20.1 μm (62 to 155 μm). The difference was statistically significant (*p* = 0.001).

**Conclusions:**

Both Visumax and Moria M2 MK are safe and effective in treating myopia with no statistically significant difference in induction of ocular aberrations but with potential advantage for Visumax regarding predictability. More accurate flap thickness is achieved with Visumax femtolasik.

**Trial registration:**

This study was retrospectively registered on 19/6/2017. Trial registration number NCT03193411, clinicalTrials.gov.

## Background

With the increasing understanding of corneal biomechanics, the pursuit of thinner and more predictable flaps led to the development of more precise MKs and to a bladeless method of flap creation [1, 2]. Good MKs are associated with accurate cuts, easy manipulation, less flap variation and fewer complications. [3] Numerous studies were reported comparing MK and femtosecond flap creation but with varying results. Some studies reported that creating LASIK flaps with the FSL resulted in better UDVA and faster visual recovery, lower postoperative astigmatism and trefoil, lesser degree of spherical aberration, faster recovery of corneal sensation, and some contrast sensitivity value [4, 5]. Others reported that the visual outcomes during the first 6 months after LASIK is not affected by the method of flap creation [6].

This prospective clinical study compared the Visumax FSL and the Moria M2 MK in treating myopia. The primary outcomes were efficacy, safety and predictability. The secondary outcomes were changes in keratometry and corneal aberration and flap thickness predictability.

## Methods

An interventional, prospective clinical study was carried out in Dar AlShifa hospiatal (Kuwait) on 60 eyes from January 2014 to June 2014. The local institutional review board approved this study. Before providing consent, all patients were given detailed information regarding each treatment. In group (I), corneal flaps were created with the Visumax FSL (Carl Zeiss Meditec, Oberkochen, Germany) while in group (II) they were created with Moria M2 MK (Moria SA, Antony, France). The patients chose whether to have the Visumax FSL or the MK to create the corneal flaps. Male and female patients were included in the study if they were older than 18 years and younger than 40 years, have stable myopia up to −6.0D and astigmatism up to −3.0D determined by manifest refraction for at least 6 months, a corrected distance visual acuity of at least 20/20, and stable keratometry after cessation of soft contact lens wear for at least 2 weeks.

Patients were excluded if they had severe dry eye, any anterior segment pathology, any form of retinal degeneration, corneal thickness that would have resulted in less than 300 μm residual stromal thickness, unstable myopia. Patients who had keratoconus or were keratoconus suspects, a history of herpes zoster ophthalmicus or herpes simplex keratitis, previous ocular surgery, a history of a steroid-responsive rise in intraocular pressure (IOP) or a preoperative IOP of more than 21 mmHg, autoimmune disease, connective tissue disease, diabetes mellitus, and chronic use of systemic corticosteroid or immunosuppressive therapy were also excluded from the study.

A complete preoperative ophthalmological examination included uncorrected (UDVA) and best corrected (CDVA) distance visual acuities using Topcon PC-50 Visual Acuity Testing System (Topcon Corp., Tokyo, Japan), determination of manifest and cycloplegic refraction (Nidek ARK-510, Japan), slit lamp biomicroscopy, Goldmann applanation tonometry, and binocular indirect ophthalmoscopy through dilated pupils. ATLAS 9000 Corneal Topography System (CarlZeiss Meditec AG, Germany) used to determine K1 (steep keratometric reading), k2 (flat keratometric reading) and Q value (corneal asphericity coefficient). Ocular aberrations were tested using a wavefront aberration-supported corneal ablation Hartmann-Shack analyzer (WASCA, Carl Zeiss Meditec AG, Germany). Wavefront examinations were done following cycloplegia with one drop of tropicamide 1% (AlconLaboratories Ltd., Hemel Hempstead, United Kingdom) and were analyzed in a 6-mm zone. Aberrations analyzed included total aberrations, high order aberrations (HOA), vertical coma, horizontal coma and spherical aberrations.

### cSurgical procedures

All patients had bilateral LASIK on the same day using the same excimer laser. The excimer laser ablation in both groups was done using The MEL-80 excimer laser (Carl Zeiss Meditec AG). This 193 nm Gaussian beam excimer laser has 1024 Hz pupil and limbal tracker that compensates for cyclotorsion and a shot frequency of 250 Hz. All surgeries were wavefront-guided and were performed by the same surgeon. The treatment was centered on visual axis and Emmetropia was aimed for all eyes. Superior-hinged flap parameters were programmed for all eyes. Treatment parameters were selected using CRS-Master software (Carl Zeiss Meditec AG), which combines refractive, wavefront, topography, and flap parameters through an interactive user interface. The ablation optical zone (OZ) diameter was selected based on the same mesopic pupil diameter obtained from the Hartmann-Shack wavefront analyzer and the software automatically calculates a transition zone up to 2.2 mm.

### FSL system

The Visumax FSL system was used to create the LASIK flap in group I. The laser uses a wavelength of 1043 nm, a repetition rate of 500 kHz, and a pulse duration of 220 to 580 femtosecond. The intended flap thickness was 100 μm. Other parameters were: flap hinge length from 3.7 to 4.0 mm; and a side cut angle of 70°. One of 3 curved contact glass sizes for the FSL was selected depending on corneal diameter measured with an Atlas topography system. The recommended minimum corneal diameter was 11.2 mm for the small contact glass, 11.7 mm for the medium contact glass, and 12.4 mm for the large contact glass.

### Mechanical MK

Corneal flaps in group II were created using The Moria M2 single-use head 90 MK with the ME-LSK evolution 3 control unit. The suction ring was chosen according to the manufacturer’s recommendations, a nomogram based on the steepest keratometric value (K1).

### Pachymetry measurements

The SP 100 Handy pachymeter (Tomey, Nagoya, Japan) which operates at 20 MHz was used for preoperative and intraoperative central corneal thickness (CCT). For preoperative measurement, One drop of 1% tetracaine hydrochloride was instilled before the pachymetric readings were obtained by a trained clinician. Excessive compression of the tip of the probe against the cornea was avoided. Three consecutive measurements were taken and the results averaged for subsequent analysis. Intraoperatively, the residual stromal thickness was measured immediately after the flap was lifted and before the ablation was performed. Again, three consecutive measurements were recorded and the results averaged. The actual flap thickness was calculated as the difference between the average preoperative CCT and intraoperative residual stromal thickness.

Postoperatively, all patients used Prednisolone acetate 1% (Pred Forte) drops 6 times daily for 7 days and tapered gradually and topical antibiotic drops (Vigamox; 0.5% moxifloxacin hydrochloride ophthalmic solution) 4 times daily for 7 days. Follow-up examinations were performed at 1 day, 1 week, 1 month, 3 months.

### Data analysis

Data analysis was performed using SPSS for Windows (version 17, SPSS Inc., Chicago). Normality was checked by the Kolmogorov-Smirnov test, and Mann–Whitney U test was performed to compare both groups. Preoperative and postoperative results were compared using Wilcoxon signed-rank test. Differences were considered to be statistically significant when *P* ≤ 0.05. Results were reported according to the standard graphs for reporting refractive surgery [7].

Outcomes parameters included: Efficacy index: ratio of postoperative UDVA to preoperative CDVA. Safety index: ratio of postoperative CDVA to preoperative CDVA. Predictability which is percentage of eyes within ±0.5D and ±1.0D of attempted correction.

## Results

This study enrolled 60 eyes of 30 patients. Patient baseline characteristics for both groups are shown in (Table 1). There were no statistically significant differences between the two groups with respect to age, manifest sphere, cylinder, spherical equivalent (SE), pachymetry, keratometry and ocular aberrations.

**Table 1:** Baseline patient characteristics

	GROUP I (30 eyes)	GROUP II (30 eyes)	*P value*
Age	26 ± 4.7 (20 to 39)	27.8 5.2 (21 to 37)	0.14
Gender: male/female	9 M/6F	8 M/7F	0.16
Preoperative manifest sphere diopter (D)	−2.5 ± 1.2 (−5.25 to −0.75)	−2.8 ± 1.01 (−5.0 to −0.5)	0.14
Preoperative cylinder (D)	−0.8 ± 0.46 (−2.0 to −0.25)	−0.6 ± 0.49 (−2.0 to −0.25)	0.13
Preoperative SE (D)	−2.45 ± 1.09 (−4.25 to −0.75)	−3.0 ± 1.07 (−5.5 to −0.5)	0.06
Preoperative k1(D)	43.9 ± 1.08 (40.8 to 45.66)	43.75 ± 1.4 (41.39 to 46.2)	0.58
Preoperative k2(D)	42.87 ± 1.15 (40.9 to 44.94)	43.07 ± 1.4 (40.44 to 45.36)	0.57
Preoperative Q(4.5 mm)	−0.38 ± 0.11 (−0.7 to −0.2)	−0.36 ± 0.11 (−0.6 to −0.1)	0.91
Preoperative pachymetry(μm)	546.71 ± 36.48 (489 to 623)	558.3 ± 40.4 (509 to 655)	0.33
Preoperative total aberrations(μm)	4.88 ± 1.6	5.2 ± 1.9	0.66
Preoperative HOA(μm)	0.37 ± 0.1	0.36 ± 0.1	0.48
Preoperative vertical coma(μm)	0.01 ± 0.2	0.02 ± 0.2	0.54
Preoperative horizontal coma(μm)	0.17± 0.4	0.2 ± 0.6	0.06
Preoperative SA(μm)	0.16 ± 0.3	0.2 ± 0.3	0.16

Abbreviations: *D* diopter, *(k1,k2)* the steep and the flat keratometric readings, *Q* corneal asphericity, *SE* spherical equivalent, *HOA* high order aberrations, μm micron *SA* spherical aberrations

### Flap thickness

In group I, the mean postoperative actual flap thickness was 100.12 ± 16.1 μm (81 to 122 μm), while in group II, it was 104.6 ± 20.1 μm (62 to 155 μm). The difference was statistically significant (*p* < 0.001).

### Efficacy

**Fig. 1:**
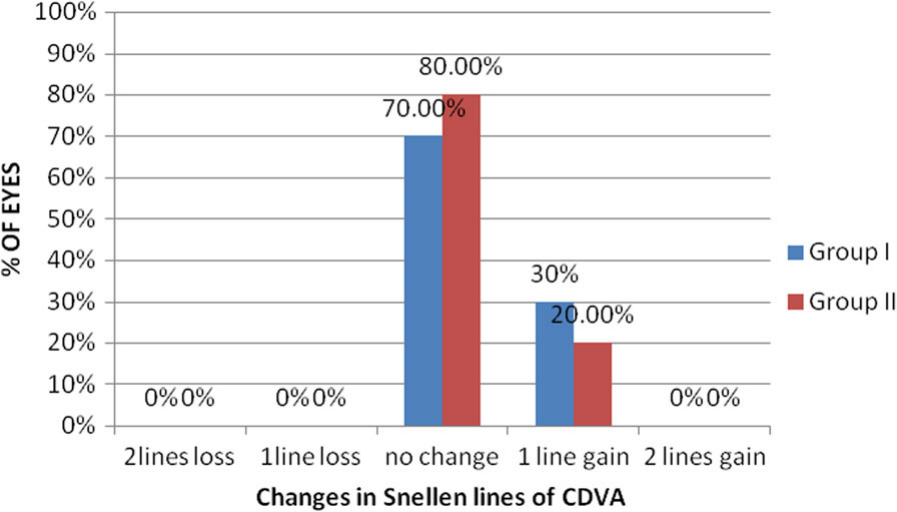
Comparison of postoperative UDVA in Visumax group (group I) and Moria M2 MK (group II)

UDVA (log MAR VA) improved from 1.17 ± 0.6 (2 to 0.4) preoperatively to −0.02 ± 0.07 (0.1 to - 0.1) at 3-month follow-up with an efficacy index of 1.06. While in group II, the UDVA (log MAR VA) improved from 1.58 ± 0.5 (2 to o.6) preoperatively to 0.01 ± 0.08 (0.3 to −0.1) with an efficacy index of 1.0. At 3 months follow up in group I, the postoperative mean manifest sphere was 0.15 ± 0.35 (−0.75 to 0.75) (*p* < 0.001), while in group II it was 0.28 ± 0.4 (−0.75 to 1.0) (*p* < 0.001). The difference in postoperative sphere between the two groups was statistically insignificant (*p* value =0.17). The postoperative cylinder was −0.37 ± 0.3 (−1.0 to 0.25) in group I (*p* = 0.00) while in group II it was to −0.33 ± 0.3 (−1.0 to 0.5) (*p* < 0.001). The difference in postoperative cylinder between the two groups was statistically insignificant (*p* value =0.94). In group I, postoperative UDVA was 20/25 in 30 eyes (100%), 20/20 in 26 eyes (86.6%) and 20/16 in 10 eyes (33.3%), while in group II, it was 20/40 in 30 eyes (100%), 20/25 in 29 eyes (96.6%), 20/20 in 24 eyes (80%) and 20/16 in 6 eyes (20%). (Figure 1). When comparing the two groups, *p* value for eyes achieving UDVA of 20/25 was 0.82, for eyes achieving UDVA of 20/20 was 0.71, and for eyes achieving UDVA of 20/16 was 0.06.

### Predictability

**Fig. 2:**
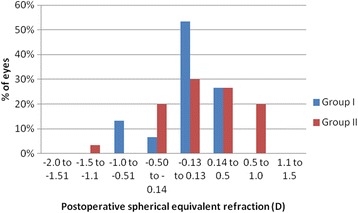
Postoperative SE refraction in Visumax group (group I) and Moria M2 MK (group II)

**Fig. 3:**
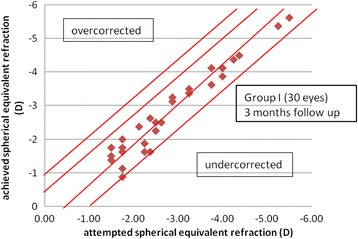
Spherical equivalent attempted versus achieved in Visumax group (group I)

**Fig. 4:**
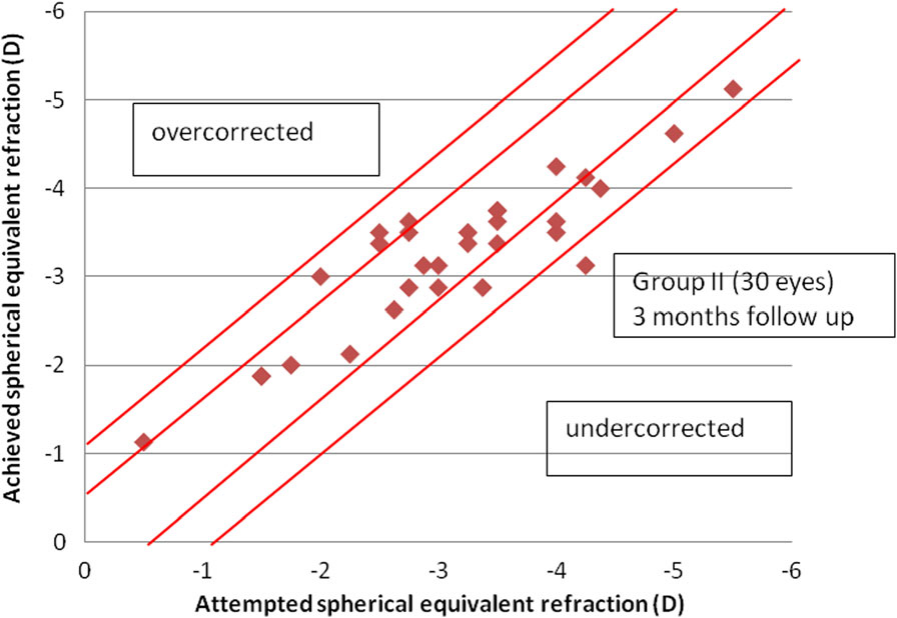
Spherical equivalent attempted versus achieved in Moria M2 MK (group II)

The mean postoperative spherical equivalent in group I was −0.02 + 0.3D (range − 0.8 to 0.3) at 3 months postoperatively, while in group II, it was 0.12 ± 0.49 D (range, −1.1 to 1). The difference in postoperative SE between the two groups was statistically insignificant (*p* = 0.21). At 3 months, all eyes in group I (100%) and 29 eyes (96.6%) in group II were within ±1D of the intended correction, 26 eyes (86.6%) in group I and 23 eyes in group II (76.6%) were within ±0.5D of the intended correction and 23 eyes (76.6%) in group I and 15 eyes in group II (50%) were within ±0.25D of the intended correction (Figs 2, 3, 4).

### Keratometry and ocular aberrations

Compared to the preoperative values, there was statistically significant change in postoperative K1, K2 and Q value (p = < 0.001, < 0.001, < 0.001respectively in group I and p = < 0.001, < 0.001, < 0.001respectively in group II). However, these changes were not statistically significant in between the two groups. Regarding ocular aberrations, all aberrations increased at 3 months postoperatively in both groups. Compared to preoperative values, there was a statistically significant increase in total aberrations, high order aberrations (HOA), vertical coma, horizontal coma and spherical aberrations. (In group I, *p* value was <0.001, 0.02, 0.02, < 0.001; and 0.02 respectively, while in group II *p* value was <0.001, 0.05, 0.03, < 0.001; and 0.01). But the difference between groups was not statistically significant as demonstrated in (Table 2).

**Table 2:** Comparison between postoperative keratometric and aberrometric changes in group I and II

	GROUP I	GROUP II	*P value*
Postoperative K1(D)	41.6 ± 1.09 (38.9 to 43.5)	41.0 ± 1.6 (38.5 to 43.6)	0.14
Postoperative K2(D)	40.7 ± 1.08 (38.8 to 42.9)	40.26 ± 1.5 (37.8 to 43.2)	0.13
Postoperative Q(4.5 mm)	0.1± 0.2 (−0.5 to 0.4)	0.2 ± 0.1 (−0.1 to 0.5)	0.07
Postoperative total aberrations(μm)	1.19 ± 0.6	1.21 ± 0.4	0.4
Postoperative HOA(μm)	0.44 ± 0.1	0.4 ± 0.1	0.46
Postoperative vertical coma(μm)	0.08 ± 0.5	0.07 ± 0.6	0.68
Postoperative horizontal coma(μm)	0.4 ± 0.6	0.3 ± 0.7	0.27
Postoperative SA(μm)	0.3 ± 0.3	0.4 ± 0.3	0.08

Abbreviations: *(k1,k2)* the steep and the flat keratometric readings, *D* diopter, *Q* corneal asphericity, *HOA* high order aberrations, μm micron, *SA* spherical aberrations

### Safety and complications

**Fig. 5:**
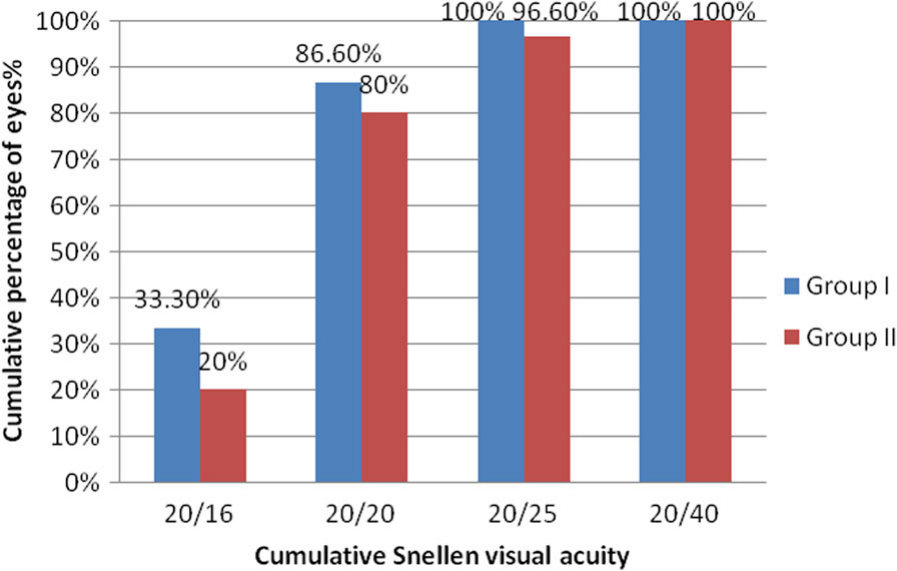
Changes in CDVA in Visumax group (group I) and Moria M2 MK (group II)

In both groups, 3 months after surgery none of the examined eyes lost any lines of CDVA. In group I, CDVA did not change in 21 eyes (70%) while 9 eyes (30%) gained 1 line. The safety index was 1.08. In group II, CDVA remained unchanged in 24 eyes (80%) while 6 eyes (20%) gained 1 line (Fig. 5). The safety index was 1.05. The difference in safety indices between the two groups was statistically insignificant (*p* = 0.3). One patient (two eyes) (6.6%) in Visumax group complained of diffuse lamellar keratitis grade II that developed on the fourth postoperative day and resolved in 2 weeks. It was treated with frequent topical steroids and resolved completely without any visual effect. One eye (3.3%) in MK group developed epithelial defect and managed with bandage contact lens. Three eyes (10%) in Visumax group and four eyes (13.3%) in MK group developed microstria and but were visually insignificant. No cases of suction loss and no cases required enhancement in both groups.

## Discussion

LASIK flaps can be created either by a mechanical MK or a FSL [8]. The recently developed, automated MKs with single use head have advantages over the traditional metallic head, such as no need for assembly, enhanced transparency and decreased risk of infection [9]. The most popular MKs for LASIK are based on the oscillating blade principle. The Moria M2 (Antony, France) is a popular compact automated MK with mechanical stop designed for maximum safety [10]. Currently, five femtosecond (FS) lasers are licensed for LASIK. [8] The move towards femtolasik is due to several advantages previously reported by this system: lower incidence of complications like epithelial abrasion, button-hole, free cap, irregular cut; greater options in flap thickness, flap diameter, hinge position and hinge length; stronger flap adherence; better contrast sensitivity; fewer induced higher order aberrations; and less incidence of dry eye [8, 11–13]. However, there are also some disadvantages of FS laser flap creation such as increased cost and larger physical size [8].

Variable results were obtained when comparing the clinical outcomes of patients who underwent LASIK with FSL versus MKs. [14, 15] To our knowledge and after going through Pubmed search; there is only one published study that compared Visumax versus Moria MK but only regarding the flap thickness predictability. [16] We choose to compare Visumax femtolaser and Moria MK due to good results obtained by the two techniques. Our results showed no significant differences between the two groups in regards to postoperative manifest sphere, astigmatism, and SE. Although there was no statistically significant difference between the two groups regarding the postoperative SE, the proportion of eyes achieving postoperative UDVA of 20/25, 20/20 and 20/16 were higher in Visumax group compared to MK group (100% versus 96.6%, 86% versus 80% and 33.3% versus 20% respectively). Moreover, the Visumax group had more patients who were within ±0.50 D and ±0.25 D of target refraction compared to the MK group (86.6% compared to 76.6% and 76.6% compared to 50% respectively). Both techniques showed acceptable safety with safety index of 1.08 in Visumax group compared to 1.05 in MK group. In addition there was no loss of lines in either group. However, the gain of one line was more pronounced in Visumax group compared to MK group (80% versus 70%).

Due to lack of availability of any published articles regarding the comparison of Visumax and Moria M2 MK, we compared our results to their previous results and our results were more or less similar to those previous reports. Huhtala A et al. [17] analyzed the results of 266 myopic eyes which were operated with Moria M2 single-use head 90 MK. After 4 weeks follow up, 92.4% of eyes were within ±0.50 D of target refraction, 97.3% eyes achieved UCVA ≥20⁄40. No eyes lost ≥2 Snellen lines. One (0.4%) eye gained 2 lines and 68 (25.8%) eyes gained 1 line. Regarding Visumax, Blum M et al. [18] treated myopia in 32 eyes with used the Visumax FSL and with the MEL 80 excimer laser. Mean preoperative SE was −4.04 +/− 1.39 diopters. After 3 months, all eyes had CDVA of 20/25 and UDVA of 20/40, 91% of eyes reached UDVA of 20/20 or better, 94% of eyes were within ±0.50 D of the planned correction. Similar results reported by Issa A et al. [19]Due to variable results obtained by comparison of different MKs and different femtolaser systems, in a recent study in 2012, Chen S et al. [14] conducted a comprehensive literature search to identify relevant trials comparing IntraLase FSL to LASIK with MKs for the correction of myopia. They found 15 articles describing a total of 3679 eyes. No significant differences were identified between the two groups in regards to patients achieving UDVA 20/20 or better, a loss of 2 lines of CDVA, final UDVA, final mean refractive SE, and final astigmatism. The IntraLase group had more patients who were within 0.50 D of target refraction compared to the MK group. One possible explanation for these findings is that the femtolaser creates uniform and accurate planar flaps rather than meniscus-shaped flaps, which are created by mechanical MKs. It was assumed that smoother optical surfaces result in better visual and refractive outcomes following laser refractive surgery [14]. Montes MR et al. [20] stated that the reason for the better results found in improved CDVA using IntraLase versus LASIK may be the decrease in use of irrigation with femtolaser. Considering that laser ablation rates vary with tissue hydration, by avoiding the need for irrigation tissue hydration may be more standardized with femtolaser than with mechanical keratomes. Another explanation given by Durrie DS et al. [5] and Kezirian GM et al. [15] was the reduction in the overall induced astigmatism in spherical treatments with the IntraLase. However, other studies were unable to prove significant differences in astigmatic refractive outcomes between the two groups. [21, 22] Only sufficient studies with a larger sample size and adequate follow-up may have the sufficient power to detect differences in predictability. Most of published articles regarding FSL are about intralase femtolaser, however recent publications proved that the efficacy, predictability, and safety profiles of the 500 kHz femtosecond platform for LASIK (Visumax system used in our study) were excellent and comparable to those of the 60 kHz platform [23].

HOA commonly increase after LASIK procedures. Recent studies have suggested that LASIK-induced aberrations might be significantly affected by the modality of flap creation. [12] It was suggested that the non-uniform flap made by the MK could disrupt the collagen fibers to a greater degree, thus, resulting in an increase in aberrations [24]. In contrast to LASIK flaps made with mechanical MKs, the geometrically planar configuration of bladeless flaps has been suggested to offer advantages over MK flaps, including the induction of fewer high-order aberrations and lesser astigmatism. [4, 12, 15] Our results show that there was statistically significant postoperative increase in HOA, total aberrations, spherical and coma aberrations in both Visumax and moicrokeratome groups but the difference between the two groups was statistically insignificant. Our results go in agreement with previous reports [14, 22]. Porter J et al. [25] found that most of the increase in spherical aberration after LASIK was due to the laser ablation and not the MK incision and the increase of coma aberrations after surgery is due to the effect of the flap hinge on the aberrations [26]. However, in another study by Montes R et al. [20], they found that FSL group showed values of the increased total, spherical, and coma like aberrations after surgery less than those of the LASIK group. They concluded that regarding spherical aberrations, the differences between the groups may be due to the use of a FSL instead of a mechanical keratome. The geometric differences created on the stromal bed between the FSL and mechanical keratome might play a role in the differences found between the two groups. Regarding coma aberrations, the flap in the microkcratome depends on the corneal diameter and corneal curvature, and the variation in the hinge angle between patients is beyond the surgeon’s control. In contrast, the hinge angle in the FSL for LASIK group is always constant. Yvon C, et al. [26] compared the change in aberrations produced by Hansatome zero-compression microkeratome and femtosecond laser (VisuMax). They found that corneas with mechanical flaps possessed significantly higher trefoil and horizontal coma. There was no change in higher-order aberrations, except for spherical aberration in the femtosecond laser group. Average change in coma did not correlate with hinge position. Both groups showed significant changes in spherical aberration. They concluded that there was greater induction of specific aberrations with the microkeratome than the femtosecond laser. Hinge position did not appear to affect the induction of coma directly, contrary to previously published reports. The difference in aberrations induction between the two groups could be due to the differences in flap thickness profiles. Differences in results between studies could arise from the different MKs or laser systems used and the degree of myopia corrected. In addition, the cornea undergoes many pathophysiological changes after LASIK, including epithelial thickening, anterior keratocytes loss, and delayed reinnervation, which may also affect outcomes [14].

Precise creation of the corneal flap is mandatory for successful LASIK. A flap that is too thin is prone to complications such as a free or buttonhole flap. A flap that is too thick may result in iatrogenic keratectasia and refractive regression. With the improvements in traditional mechanical MK systems and the development of the FSL, it is now possible to create clinically uniform flaps in LASIK [27]. Mechanical MK were known to have a low level of precision in creating corneal flaps of accurate thickness and have typically demonstrated a standard deviation of flap thickness between 18 and 24 μm [28]. Others reported standard deviation of flap thickness achieved by mechanical MK in the range of ±20 to ±40 μm. [29] Recent studies found that the FSL created flaps show less variability in flap thickness to be within ±20 μm of the intended result [29, 30]. Our results show that in Visumax group, the mean postoperative actual flap thickness was 100.12 ± 16.1 μm (81 to 122 μm) while in M2 moria MK group, it tended to cut less than the manufacture expected (120 μm) and with wide range. The mean postoperative actual flap thickness was 104.6 ± 20.1 μm (62 to 155 μm). The difference was statistically significant (*p* = 0.001). Our results go in agreement with previous reports. Huhtala A et al. [17] measured the corneal thickness of 266 myopic eyes by ultrasonic pachymetry and Moria M2 single-use head 90 was used to create a flap with a thickness of 120 μm. Mean corneal flap thickness was 115.4 ± 12.5 μm (73–147 μm). They concluded that as with most MKs, the single-use head 90 MK cut thinner flaps than were intended. The range of the cuts was relatively wide. However, thin flaps did not increase the rate of flap-related complications. Undercutting (i.e. cutting corneal flaps that are thinner than intended) appears to be the most common problem with most MKs other than the Amadeus. Thin flaps are more liable to buttonholes and cause more complicated handling during surgery. [17] Although the M2 single use head tends to cut less than the manufacture expected, its cuts showed smaller variability comparable not only to other Moria heads but also to other MKs with different heads [31]. Literature reports have been published on the Intralase and Visumax showing that these FSLs have better flap thickness predictability and better visual outcomes than mechanical MKs [13, 32]. Reinstein DZ et al. [33] assessed the accuracy and reproducibility of central flap thickness for flaps created with the VisuMax FSL in 24 eyes of 12 myopic LASIK patients with intended central flap thickness of 110 microm. Artemis 1 very high-frequency digital ultrasound scans were performed preoperatively and 3 months postoperatively. 3 months postoperatively, mean central flap thickness was 112.3+/−7.9 microm (range: 102.6 to 132.9 microm). The accuracy was 2.3 microm and reproducibility was 7.9 microm.

Issa A et al. [19] measured flap thickness by ultrasound pachymetry in 195 eyes treated with Visumax. The mean achieved flap thickness was 109.94 ± 13.43 μm (attempted 100 μm). One of the limitations in our study is the use of ultrasound subtraction pachymetry. Although it is considered the most popular method for calculating intraoperative flap thickness in clinical practice, it has potential pitfalls including the risk of infection transmission with the ultrasound probe, creating localized variations in corneal bed hydration, which could affect the laser ablation, low precision because the measurements are repeated once on the surface and another time on the bed; it is difficult to superimpose the two and the interval between femtosecond bubble creation and taking the measurement.[1] Despite this, in our clinical study, ultrasound pachymetry provided good evidence that flaps produced with the Visumax FSL had a better SD and lower variability. This is confirmed by results in other studies, which found a lower SD of flap thickness with FSLs than with mechanical MKs using different techniques and instrumentation.[34] In addition, optical coherence tomography shows that FSLs create more planar flaps with precise flap architecture, especially in the peripheral area [16].

As with any other surgical procedure, complications can occur in LASIK surgery. The incidence of flap-related complications in previous LASIK series is reported to be between 0.3 and 14% and depends on the type of the MK used and the surgeon involved.[35] In our study, one eye (3.3%) in MK group developed epithelial effect. The overall incidence of epithelial defects during LASIK is thought to be between 2.6 and 14%. Different MKs have different rates of associated epithelial defects. [36] Corneal epithelial defects during LASIK result from trauma by the corneal markers, the suction ring, overuse of topical anesthetic drops, passage of the MK over the surface, dehydration and drying of the flap, or minor trauma by forceps or spatula. An epithelial defect may increase the patient’s risk of developing DLK, flap striae, and epithelial ingrowth.[37] The incidence of epithelial defect is lower with FSL than with MKs. Moshirfar M et al. [38] reported that the MK group had a significantly greater number of epithelial defects (2.6%) than the IntraLase group (0.6%) as the FSL requires no direct shearing force on the corneal surface, whereas the MK pivots the keratome head across the corneal epithelium under high pressure. In Visumax group, one patient (two eyes) (6.6%) complained of diffuse lamellar keratitis (DLK) grade II. The challenge of FSL is to deliver the enough energy to the cornea to allow adequate resection. Too much energy may lead to undesirable effects including opaque bubble layer formation, DLK, and transient light sensitivity, whereas too little energy can result in uneven lamellar resection and difficult flap lift. [28] DLK was relatively common after initial FS laser surgeries as a result of the more intense inflammatory response generated with the early models. [12] More recent models, such as the 60 kHz IntraLase and 200 kHz Visumax, induce less inflammatory response that is similar to that with MKs. [39] Our results regarding complications are similar to those reported in Chen’s systematic review and meta-analysis, who found that the MK group had more epithelial defects, whereas the IntraLase group had more cases of DLK [14].

## Conclusion

Our study showed that both Visumax and Moria M2 MK are safe and effective in treating myopia with potential advantage for Visumax regarding predictability. More accurate flap thickness is achieved with Visumax femtoLASIK.

## Abbreviations

(k1,k2): The steep and the flat keratometric readings
D: Diopter
FSL: Femtoseconad laser
HOA: High order aberrations
MK: Microkeratome
Q: Corneal asphericity
SA: Spherical aberrations
SE: Spherical equivalent
